# Palmitate Stimulates Expression of the von Willebrand Factor and Modulates Toll-like Receptors Level and Activity in Human Umbilical Vein Endothelial Cells (HUVECs)

**DOI:** 10.3390/ijms25010254

**Published:** 2023-12-23

**Authors:** Agnieszka K. Seliga, Krzysztof Zabłocki, Joanna Bandorowicz-Pikuła

**Affiliations:** Laboratory of Cellular Metabolism, Nencki Institute of Experimental Biology PAS, 3 Pasteur Str., 02-093 Warsaw, Poland; a.seliga@nencki.edu.pl (A.K.S.); k.zablocki@nencki.edu.pl (K.Z.)

**Keywords:** endothelium, palmitate, von Willebrand factor, NF-κB, TLR2, TLR4

## Abstract

An increased concentration of palmitate in circulation is one of the most harmful factors in obesity. The von Willebrand factor (vWF), a protein involved in haemostasis, is produced and secreted by the vascular endothelium. An increased level of vWF in obese patients is associated with thrombosis and cardiovascular disease. The aim of this study was to investigate a palmitate effect on vWF in endothelial cells and understand the mechanisms of palmitate-activated signalling. Human umbilical vein endothelial cells (HUVECs) incubated in the presence of palmitate, exhibited an increased *VWF* gene expression, vWF protein maturation, and stimulated vWF secretion. Cardamonin, a Nuclear Factor kappa B (NF-κB) inhibitor, abolished the palmitate effect on *VWF* expression. The inhibition of Toll-like receptor (TLR) 2 with C29 resulted in the TLR4 overactivation in palmitate-treated cells. Palmitate, in the presence of TLR4 inhibitor TAK-242, leads to a higher expression of *TLR6*, *CD36*, and *TIRAP*. The silencing of TLR4 resulted in an increase in TLR2 level and vice versa. The obtained results indicate a potential mechanism of obesity-induced thrombotic complication caused by fatty acid activation of NF-κB signalling and vWF upregulation and help to identify various compensatory mechanisms related to TLR4 signal transduction.

## 1. Introduction

The vascular endothelium, a single layer of squamous endothelial cells lining the interior surface of blood and lymphatic vessels, is dynamically regulated and crucial in maintaining vascular homeostasis. It contributes to the regulation of the body’s response to pro-inflammatory and pro-thrombotic signals as it regulates the adhesion and aggregation of platelets, coagulation, fibrinolysis, trafficking of white blood cells, as well as innate and acquired immune responses [1]. Endothelial dysfunction that is evident by an increased vascular permeability, an impairment of NO-dependent function, and both pro-thrombotic and pro-inflammatory phenotypes is a common feature of most cardiovascular diseases. An inappropriate diet, a sedentary way of life, and a lack of physical activity result in obesity, insulin intolerance, diabetes, and multiple damages to a variety of organs and tissues, including the vascular endothelium. The endothelium is the first barrier that isolates deeper-located tissues from the blood transporting not only oxygen, carbon dioxide, nutrients, and other vital compounds, but also toxins, bacteria, lipoproteins, and free fatty acids (FFAs) complexed with proteins that may exert different harmful effects on vascular physiology [2]. In healthy individuals, the approximate concentration of FFAs in the circulation is as high as 400 μM, and can increase above 600 μM in obese individuals or those with type 2 diabetes [3,4,5] as a result of an increased intake of saturated fatty acids or lipogenesis [6]. An elevated blood plasma concentration of fatty acids impairs endothelium-dependent dilation [7,8]. Moreover, some saturated fatty acids increase the risk of cell apoptosis, leading to cardiovascular diseases [9]. The detrimental potency of free fatty acids is unequal, but palmitate (PA) is particularly harmful to the endothelium [10]. Palmitic acid is the most abundant saturated fatty acid (SFA) in circulation and represents 27.8% of the total plasma FFAs and 64% of the saturated FFAs [11]. Non-esterified palmitic acid was found to affect endothelial mitochondrial function and its in vitro application at relatively low concentrations (above 200 μM) led to cell death [12]. Thus, the effects of fatty acids (palmitate in particular) have been broadly described by others, but those studies were mainly focused on endothelial functions closely related to vasodilatation, the regulation of blood flow, inflammatory response, and cell death. Previously, we found that the mild treatment of EA.hy926 cells with palmitate resulted in a pro-survival response rather than cell death [13]. Here, our interest is focused on the less elaborated effect of palmitate, which is the endothelial secretory activity, with special attention to the von Willebrand factor (vWF). It is noteworthy that vWF synthesis and release are related to inflammation and thrombosis [14], and were found to be seriously affected in endothelial cells upon treatment with LPS (lipopolysaccharide) and TNF-α [15,16]. It is also affected in obese patients with cardiovascular disease [17,18]. However, the effects of fatty acids in this context have not been investigated. Obesity may have multiple aetiology, thus an investigation of this pathology in humans or animals in vivo could be very difficult because of the high complexity of these models. Therefore, we tested the effects of palmitate on HUVECs, which are commonly used in studies focused on endothelial metabolism. We hypothesised that palmitate at a concentration sufficient to induce a proinflammatory response of the cell but not high enough to induce cell death may influence vWF production and/or secretion. vWF is a multimeric plasma glycoprotein synthesised and released by megakaryocytes and the vascular endothelium. This multi-domain protein undergoes extensive intracellular processing in the Golgi apparatus, including cleavage by furin convertase, glycosylation, and multimerisation, and is packed into Weibel–Palade bodies (WPBs) [19]. After such modifications, vWF plays a role in the regulation of blood clotting when secreted into circulation. Thus, it may be supposed that vWF maturation subjects to multi-stage regulation.

Palmitate interaction with cells is mediated by a few specialised proteins [20]. Among them, there are Toll-like receptors (TLRs), which are pattern recognition receptor (PRR) components of the innate immune system. TLR signalling stimulates intracellular signalling cascades, depending on the ligand, activated receptor, and the adaptor molecule involved. MyD88-dependent pathway leads to the production of proinflammatory cytokines by Nuclear Factor kappa B (NF-κB) activation [21]. TLR activation affects vascular function and remodelling. Chronic low-grade inflammation caused by prolonged or excessive activation of TLRs results in endothelial dysfunction [22].

Obesity-related inflammation is possibly induced by TLR2 and TLR4 activation [23]. Moreover, TLR2 and TLR4 are involved in SFA-induced inflammation [24]. Both receptors were found to be involved in alterations observed in response to palmitate and high-fat-diet mice models [25,26,27,28,29,30,31,32,33,34,35,36,37].

Here, we report that palmitate-caused effects in HUVECs are related to TLR4. Moreover, we found a compensatory mechanism of TLR signalling regarding TLR4 inhibition, as well as TLR2 and TLR4 mRNA silencing.

## 2. Results

### 2.1. Palmitate Induces Inflammatory Response and Elevates vWF-Encoding Gene Expression and Release

HUVECs incubated with palmitate exhibit concentration- and time-dependent elevations of ICAM-1 and VCAM-1 protein levels.

**Figure 1:**
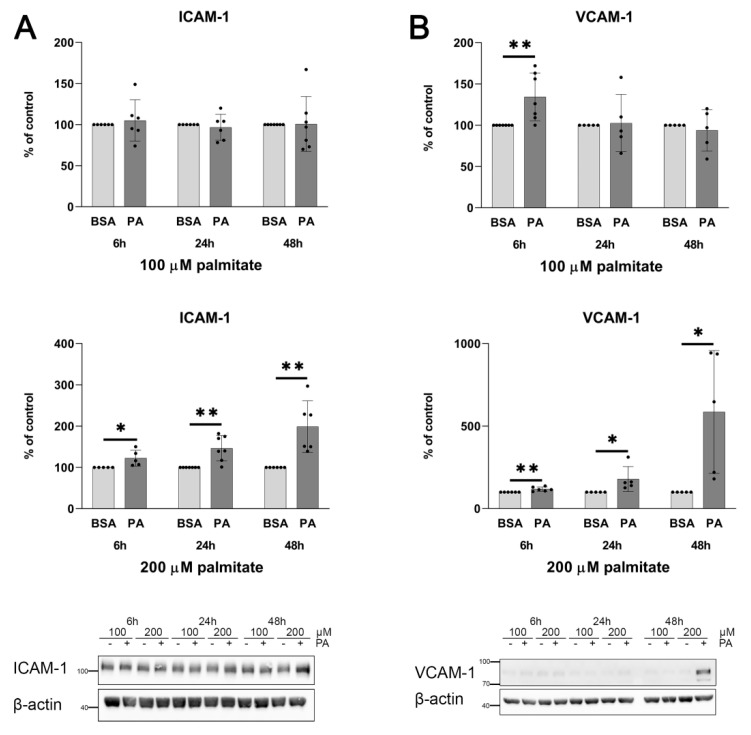
Effect of palmitate on ICAM-1 or VCAM-1 relative protein levels. Quantitative analysis and representative Western blots of (**A**) ICAM-1 and (**B**) VCAM-1. Relative protein expression normalised to β-actin and compared to BSA treated as a control. Data are expressed as a percentage of appropriate BSA control and presented as mean ± SD (n = 5–7); * *p* ≤ 0.05, ** *p* ≤ 0.01.

**Figure 2:**
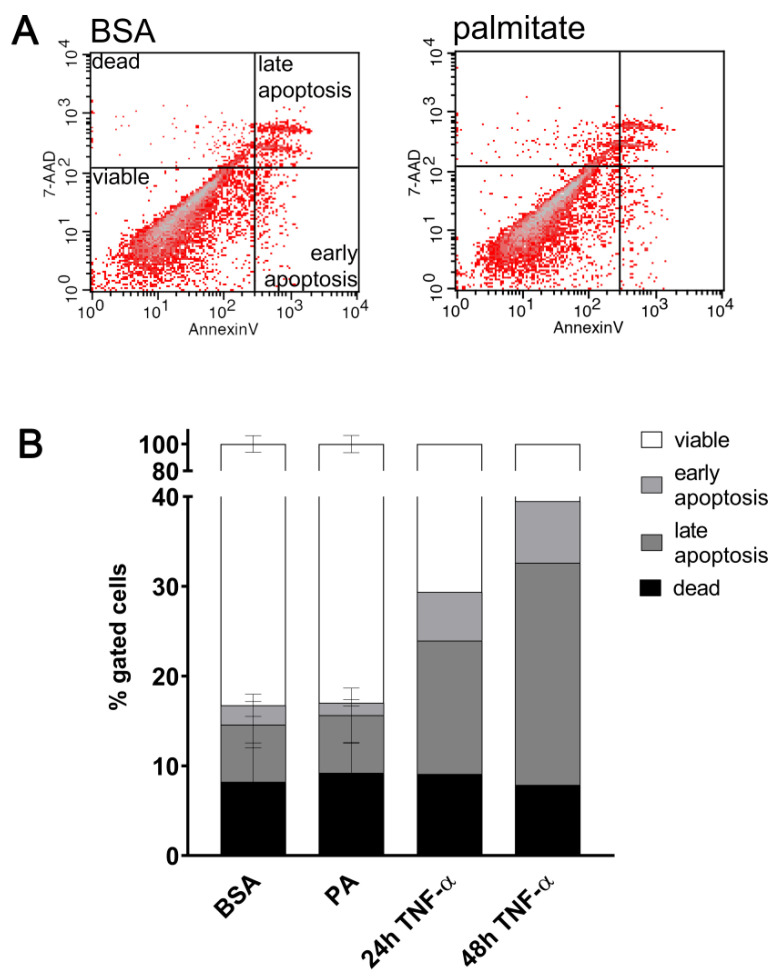
Effect of palmitate on HUVECs’ viability. Flow cytometric analysis of apoptosis with PE Annexin V and 7-AAD staining. HUVECs were treated 48 h with 200 μM palmitate or BSA as a control. (**A**) Representative histogram of flow cytometric analysis. Q1, dead cells; Q2, late apoptotic cells; Q3, viable cells; Q4, early apoptotic cells. (**B**) Quantitative analysis. Data are presented as mean value of a percentage of gated cells ± SD (n = 4). Cells were treated for 24 or 48 h with 10 ng/mL TNF-α as a control of apoptosis induction.

In all experiments shown here, palmitate has been used at a concentration high enough to elevate a level of adhesive molecules (Figure 1) but insufficient to reduce cell viability (Figure 2).

**Figure 3:**
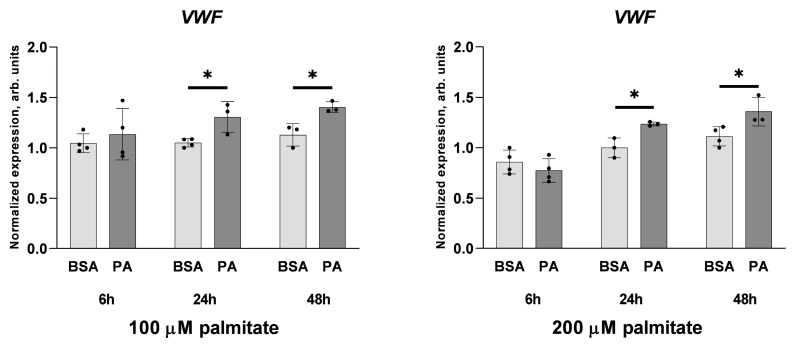
Effect of palmitate on *VWF* gene expression. Relative *VWF* gene expression after incubation with 100 or 200 μM palmitate, respectively, for the indicated time. Data analysed via the 2^−ΔΔCT^ method are presented as mean ± SD (n = 3–4); * *p* ≤ 0.05.

Under such conditions, a level of *VWF* transcript was noticeably elevated upon treatment of cells with palmitate (vs. control cells) regardless of whether it was applied at 100 or 200 μM concentration (Figure 3).

**Figure 4:**
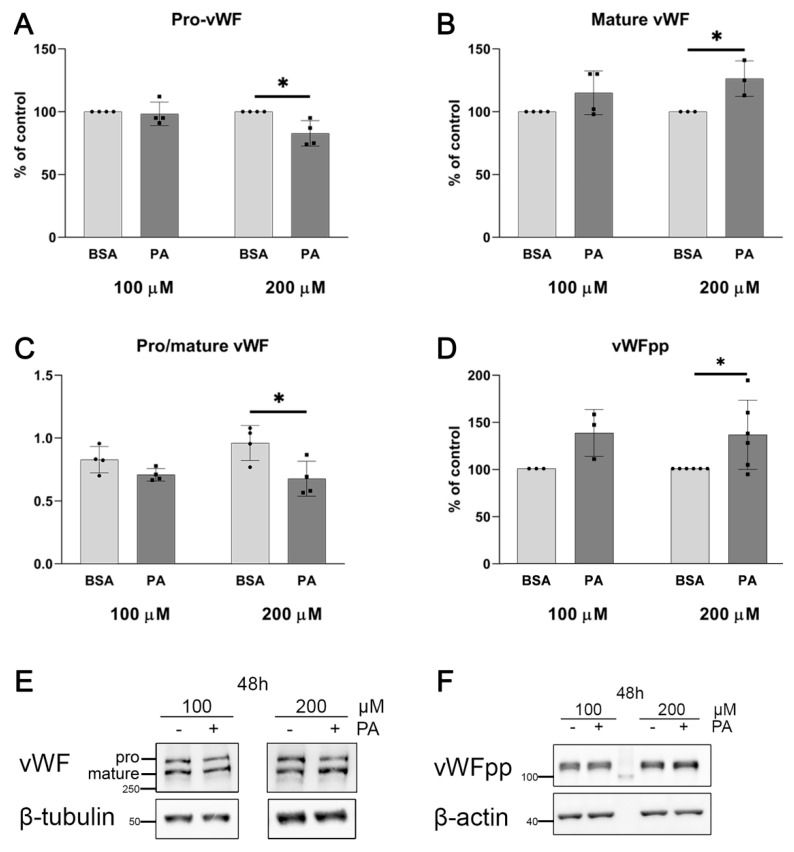
Effect of palmitate on relative von Willebrand factor (vWF) protein level. Quantitative analysis of relative protein expression: (**A**) pro-vWF; (**B**) mature vWF; (**C**) ratio of pro-vWF to mature vWF; (**D**) vWFpp normalised to β-actin or β-tubulin and compared to BSA treated as a control. Data are expressed as a percentage of appropriate BSA control and presented as mean ± SD (n = 3–6); * *p* ≤ 0.05. Representative Western blots of (**E**) pro- and mature vWF and (**F**) vWFpp.

Two products of a post-translational modification of the precursor vWF protein (pro-vWF), which are the mature vWF with haemostatic function and the much shorter vWF antigen 2, also known as vWF propeptide (vWFpp) (do not confuse with pro-vWF, which is the longest primary product of translation) with biological activity [38], are stored in WPBs and released from cells [39]. Figure 4 shows the proportions of different forms of vWF, which are affected in cells treated with palmitate. Moreover, an increased intracellular level of the mature vWF and vWFpp upon treatment of cells with palmitate suggests an elevated gene expression and/or stimulated protein maturation (Figure 4).

**Figure 5:**
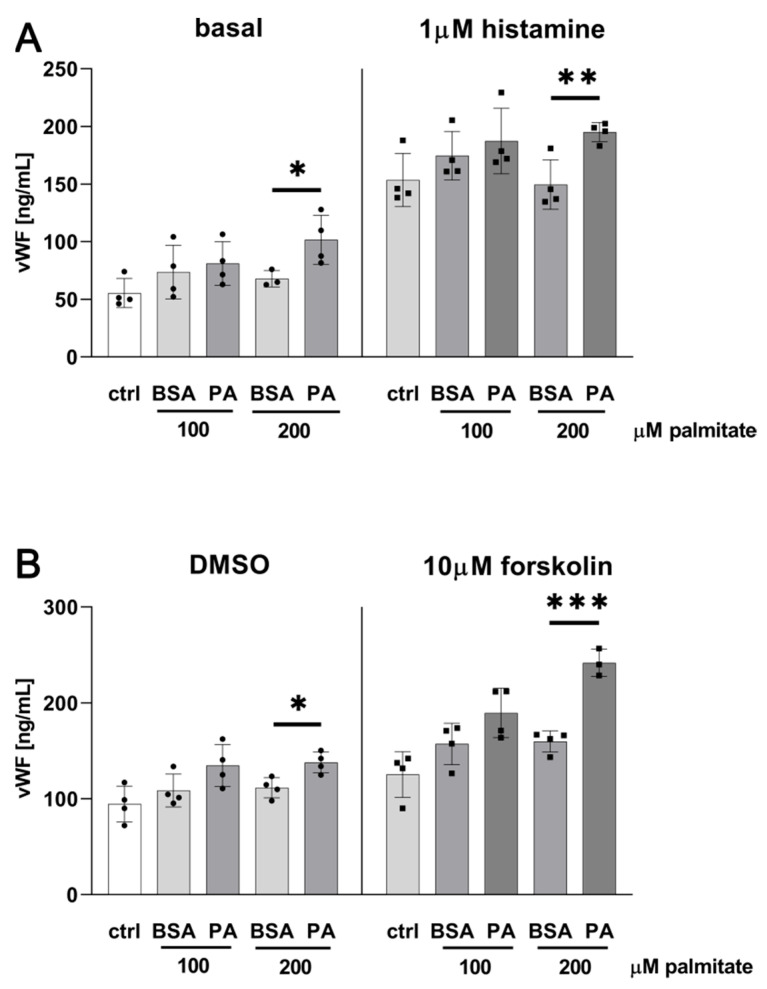
Secretion of von Willebrand factor. HUVECs incubated 48 h in the presence of 100 or 200 μM palmitate were stimulated with (**A**) 1 μM histamine or (**B**) 10 μM forskolin (DMSO as control). Basal or stimulated vWF secretion was measured via ELISA. Data are presented as mean ± SD (n = 3–4); * *p* ≤ 0.05, ** *p* ≤ 0.01, *** *p* ≤ 0.001.

As shown in Figure 5, an incubation of HUVECs in the presence of 200 μM palmitate for 48 h results in a moderate but statistically significant elevation of histamine- or forskolin-induced vWF secretion to the extracellular fluid, while the stimulatory effect of palmitate alone is less notable. Palmitate applied at 100 μM concentration seems to be insufficient in this matter despite the fact of an elevated *VWF* transcript content identified with the use of qPCR (Figure 3).

**Figure 6:**
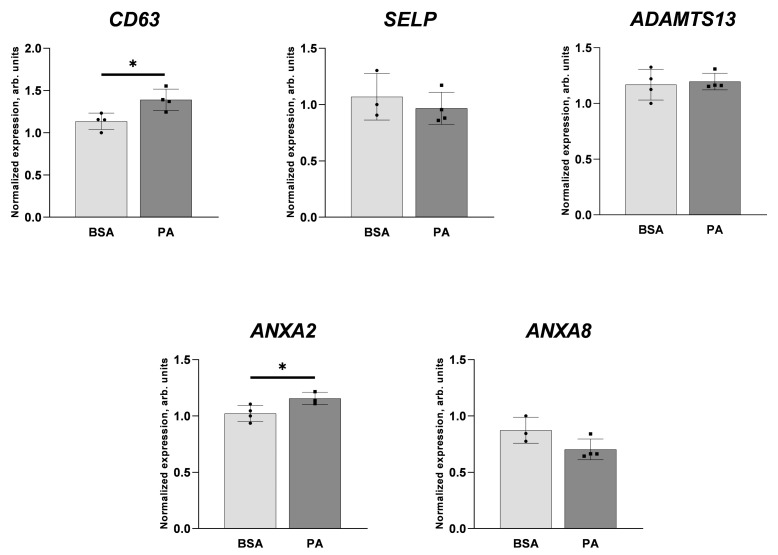
Effect of palmitate on *CD63, SELP, ADAMTS13, ANXA2,* and *ANXA8* gene expression. Relative gene expression after incubation with 200 μM palmitate for 48 h. Data analysed using the 2^−ΔΔCT^ method are presented as mean ± SD (n = 3–4); * *p* ≤ 0.05.

Figure 6 shows that palmitate exerts only a slight—if any—effect on the transcript levels of selected genes encoding proteins related to vWF biology, i.e., involved in the secretion or maturation of vWF. Palmitate upregulates the mRNA level of *CD63* and *ANXA2*, without an effect on *SELP* and *ANXA8*. These results do not confirm a putative effect of palmitate on the WPBs and suggest that the palmitate-induced elevation of the vWF release after treatment by histamine or forskolin reflects an increased expression of the vWF-encoding gene and maturation of vWF protein. Moreover, palmitate does not affect the level of *ADAMTS13* mRNA.

**Figure 7:**
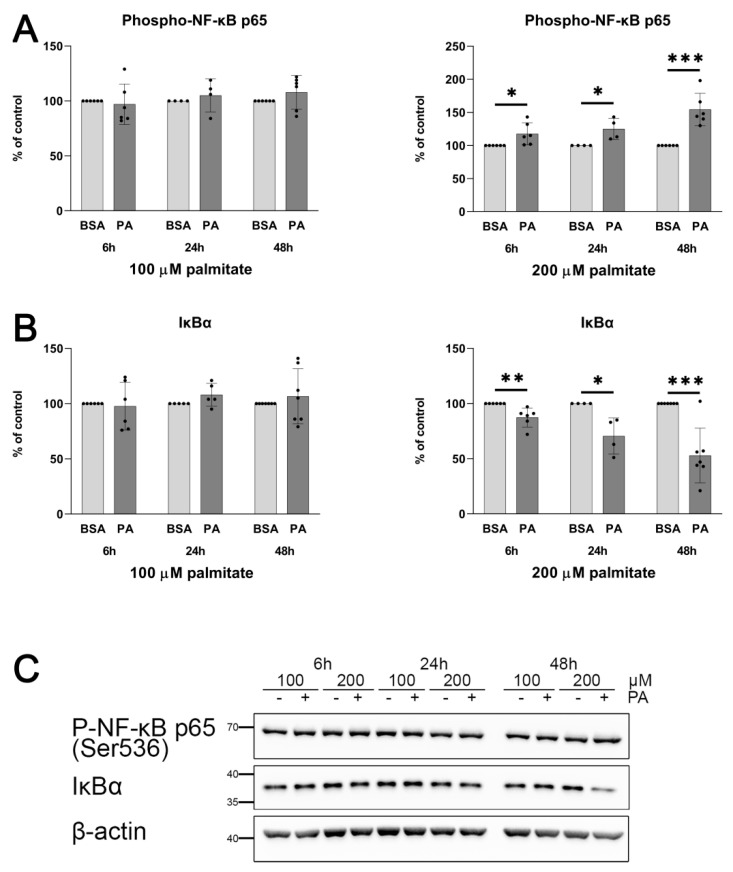
Effect of palmitate on relative phospho-NF-κB p65 (Ser536) and IκBα protein levels. Quantitative analysis of (**A**) phospho-NF-κB p65 and (**B**) IκBα relative protein expression normalised to β-actin and compared to BSA treated as a control. Data are expressed as a percentage of appropriate BSA control and presented as mean (n = 4–7); * *p* ≤ 0.05, ** *p* ≤ 0.01, *** *p* ≤ 0.001. (**C**) Representative Western blots.

To identify and follow a signalling cascade that is mobilised in the palmitate-treated HUVECs and that leads to the elevation of vWF secretion, we focused our attention on the NF-κB pathway, which was previously found by other authors to be activated in the palmitate-treated endothelial cells [40]. Figure 7 confirms that the incubation of HUVECs with 200 μM palmitate resulted in a simultaneous decrease in the IκBα level and an increase in the proportion of the phosphorylated form of NF-κB p65.

**Figure 8:**
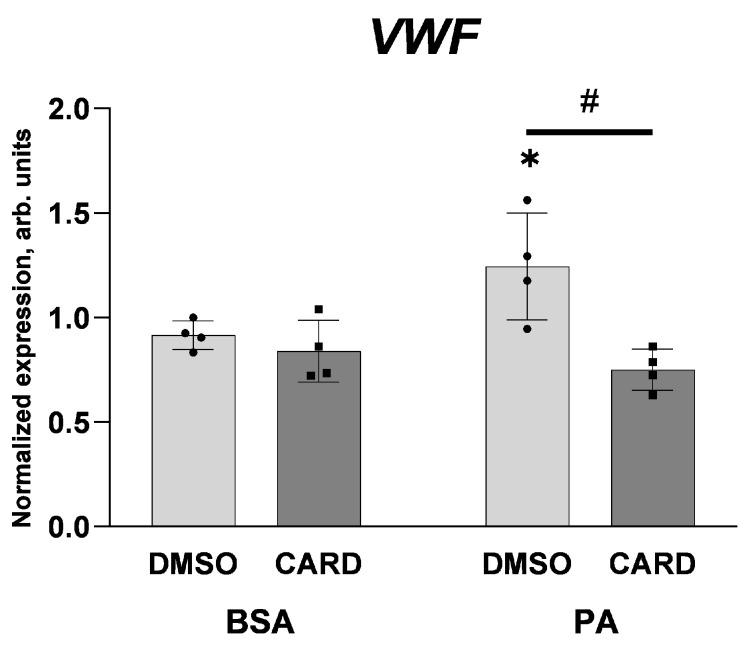
Effect of cardamonin and palmitate of *VWF* gene expression. Relative *VWF* gene expression after 48 h co-incubation of 100 μM palmitate with 5 μM cardamonin. Data analysed via the 2^−ΔΔCT^ method are presented as mean ± SD (n = 4); * *p* ≤ 0.05 PA vs. BSA; # *p* ≤ 0.05 inhibitors vs. DMSO.

It is noteworthy that the inhibition of NF-κB transcription factor with cardamonin completely abolishes the stimulatory effect of palmitate on *VWF* gene expression (Figure 8). Because 100 μM palmitate was sufficient to elevate vWF-encoding mRNA (see Figure 4) in the case shown in Figure 8, the same experimental conditions were applied. Interestingly, in cells treated with 100 μM palmitate, no stimulatory effect on NF-κB activation visualised as increased NF-κB p65 phosphorylation or decreased IκBα levels was observed (Figure 7) and a cellular level of adhesion proteins was not affected either (Figure 1). However, the sensitivity to cardamonin expressed as a reduced *VWF* gene transcription seems to indicate NF-κB contribution.

### 2.2. Palmitate Affects TLR Levels and Activity

**Figure 9:**
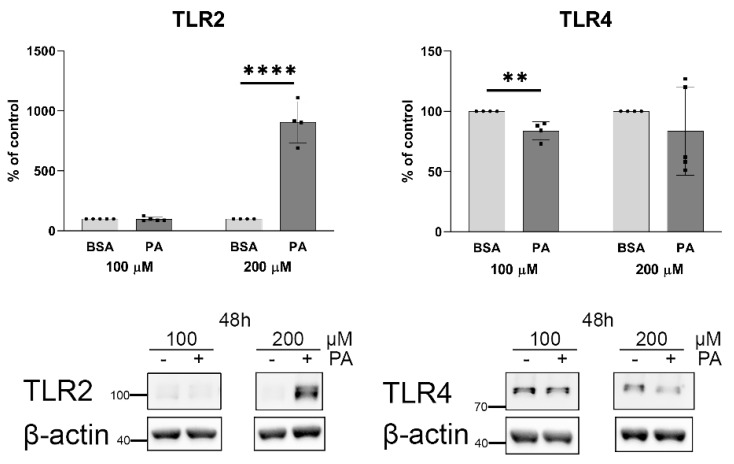
Effect of palmitate on TLR2 and TLR4 relative protein levels. Representative Western blots and quantitative analysis of relative protein expression normalised to β-actin and compared to BSA treated as a control. Data are expressed as a percentage of appropriate BSA control and presented as mean ± SD (n = 4–5); ** *p* ≤ 0.01, **** *p* ≤ 0.0001.

The stimulation of NF-κB signalling pathways by palmitate and the role of this transcription factor were described earlier [40,41,42] (although in another context), but cellular receptors involved in this response are still questionable [20,43]. As shown in Figure 9, an incubation of HUVECs with 200 μM palmitate results in an unexpectedly huge increase in TLR2 protein levels while TLR4 remains unaffected (or even decreased) under such conditions.

**Figure 10:**
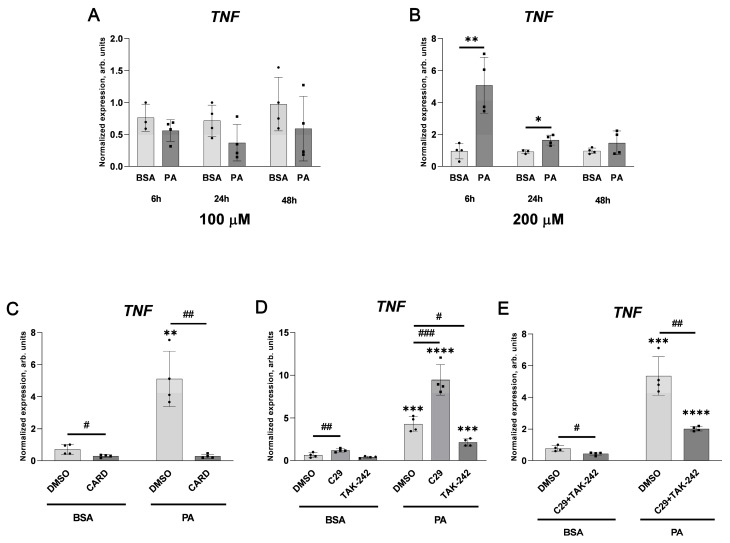
Effect of palmitate, cardamonin, and TLR inhibitors on the *TNF* transcript level. Relative *TNF* gene expression after incubation with (**A**) 100 μM and (**B**) 200 μM palmitate, respectively, for the indicated time, 6 h co-incubation of 200 μM palmitate with inhibitors: (**C**) 20 μM cardamonin (CARD); (**D**) 100 μM C29, 20 μM TAK-242; (**E**) both 100 μM C29 and 20 μM TAK-242 simultaneously. Data analysed using the 2^−ΔΔCT^ method are presented as mean ± SD (n = 4); * *p* ≤ 0.05, ** *p* ≤ 0.01, *** *p* ≤ 0.001, **** *p* ≤ 0.0001 PA vs. BSA; # *p* ≤ 0.05, ## *p* ≤ 0.01, ### *p* ≤ 0.001 inhibitors vs. DMSO.

As shown in Figure 10, under the same experimental conditions, 200 μM palmitate transiently elevated *TNF* transcript levels and this effect, observed after 6 h of incubation, was much stronger than after 24 h of the treatment.

The almost complete inhibitory effect of cardamonin confirms the role of NF-κB in activating the TNF-α-encoding gene in palmitate-treated cells. Surprisingly, inhibition of TLR2 with compound C29 resulted in a strong elevation in the *TNF* transcript level, while TAK-242, which inhibits TLR4 activity, reduced palmitate-stimulated *TNF* gene transcription. The effect of both compounds added simultaneously was also inhibitory in comparison to the effect of DMSO added as a solvent for both inhibitors. These data suggest that the inhibition of TLR2 activates the compensatory response of alternative pathways.

**Figure 11:**
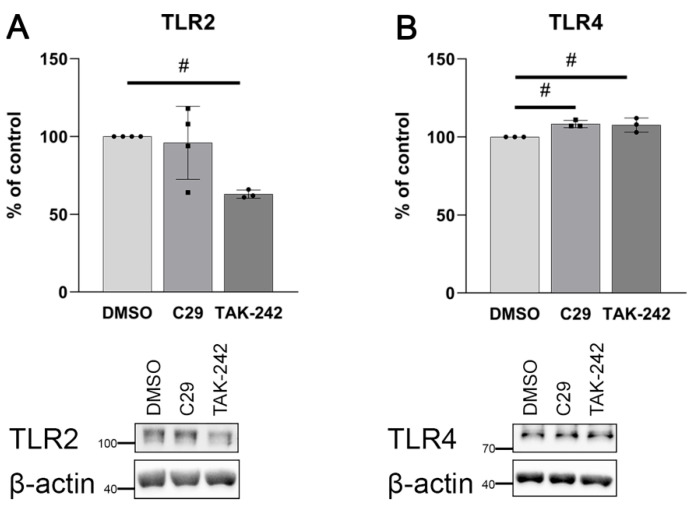
Effect of TLR inhibitors on TLR2 and TLR4 relative protein levels. HUVECs were treated 7 h with 100 μM C29 and 20 μM TAK-242. Quantitative analysis and representative Western blots of (**A**) TLR2 and (**B**) TLR4. Relative protein expression normalised to β-actin and compared to DMSO treated as a control. Data are expressed as a percentage of DMSO control and presented as mean ± SD (n = 3–4); # *p* ≤ 0.05 inhibitors vs. DMSO.

Indeed, Figure 11 confirms a cross-talk between TLR2 and TLR4 in cells incubated with C29 and TAK-242, but only a very slight effect of C29 on TLR4 protein content hardly justified the elevation of the *TNF* transcript level.

**Figure 12:**
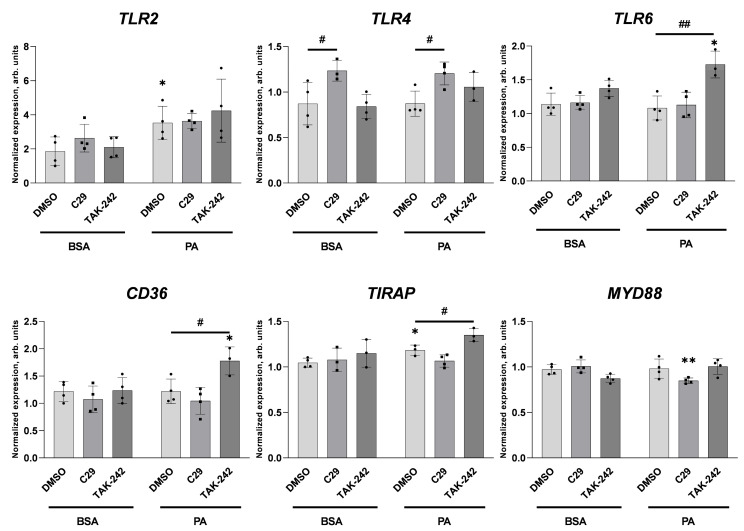
Effect of C29 and TAK-242 on gene expression of *TLR2, TLR4,* and *TLR6* and proteins participating in TLRs activity. Relative *TLR2, TLR4, TLR6, CD36, TIRAP,* and *MYD88* gene expression after 6 h co-incubation of 200 μM palmitate with inhibitors: 100 μM C29 and 20 μM TAK-242. Data analysed using the 2^−ΔΔCT^ method are presented as mean ± SD (n = 3–4); * *p* ≤ 0.05, ** *p* ≤ 0.01 PA vs. BSA; # *p* ≤ 0.05, ## *p* ≤ 0.01 inhibitors vs. DMSO.

To investigate the compensatory mechanism in palmitate-treated cells exposed to C29 and TAK-242, we tested the gene expression of various TLR-related proteins. We found that palmitate increased *TLR2* expression. Consistently with its effect on TLR4 protein levels (Figure 11), inhibitor C29 increased *TLR4* expression regardless of the presence of palmitate (Figure 12). Moreover, we found that palmitate incubation in cells pre-treated with TLR4 inhibitor resulted in increased *TLR6, CD36,* and *TIRAP* expression levels (Figure 12). It suggests the compensatory effect of TLR4-interacting proteins caused by an activation of the inhibited TLR4 receptor.

**Figure 13:**
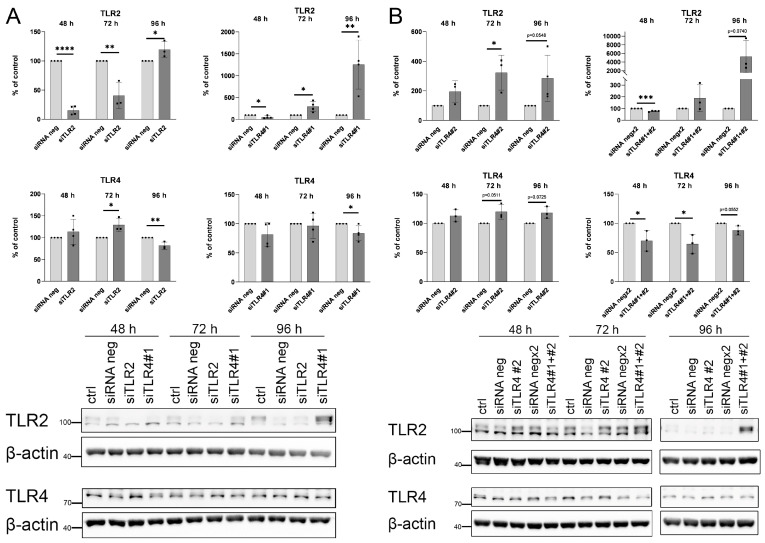
Effects of TLR2 and TLR4 gene silencing on protein levels of these receptors. HUVECs transfected with siTLR2 and siTLR4 were lysed 48, 72, and 96 h post-transfection. (**A**) Quantitative analysis and representative Western blots of TLR2 and TLR4 in cells transfected with siTLR2 or siTLR4#1. (**B**) Quantitative analysis and representative Western blots of TLR2 and TLR4 in cells transfected with siTLR4#2 or siTLR4#1 and siTLR4#2. Relative protein expression normalised to β-actin and compared to siRNA negative-control (siRNA neg) transfected cells as a control or siRNA negx2 for cells transfected with two siRNA. Data analysed using the 2^−ΔΔCT^ method are expressed as a percentage of siRNA negative control and presented as mean ± SD (n = 3–4); * *p* ≤ 0.05, ** *p* ≤ 0.01, *** *p* ≤ 0.001, **** *p* ≤ 0.0001.

To validate the mutual compensatory effect of TLR2 and TLR4, protein synthesis was inhibited by silencing RNA. A reduced TLR2 level resulted in a transient increase in TLR4 protein (Figure 13A). Transfection with siTLR4#1 caused a mild TLR4 protein decrease, but caused a substantial TLR2 increase (Figure 13A). Transfection with different siRNA specific for TLR4 siTLR4#2 also resulted in TLR2 increase despite the fact that TLR4 protein level was unaffected or even increased (Figure 13B). Transfection with two siRNA siTLR4#1 and siTLR4#2 at the same time resulted in an efficient decrease in TLR4 protein level and an enormous TLR2 increase.

**Figure 14:**
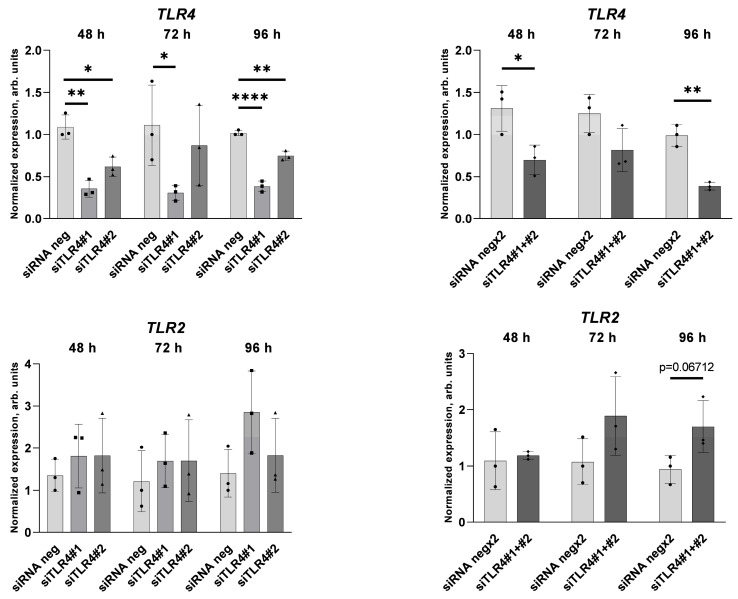
Effect of the TLR4 gene silencing on *TLR4* or *TLR2* gene expression. Relative *TLR4* or *TLR2* gene expression in HUVECs transfected with siTLR4#1, siTLR4#2, or both siTLR4#1 and siTLR4#2 were analysed 48, 72, and 96 h post-transfection. Data analysed using the 2^−ΔΔCT^ method are presented as mean ± SD (n = 3); * *p* ≤ 0.05, ** *p* ≤ 0.01, **** *p* ≤ 0.0001.

TLR4 silencing was confirmed on the transcript level. *TLR4* gene expression decreased in cells transfected with both siRNA specific for TLR4 applied separately and transfected with two siRNA applied together (Figure 14). The effect of siTLR4 transfection on *TLR2* gene expression is not significant, although there is a tendency to increase *TLR2* expression.

## 3. Discussion

Previous studies on palmitate effects in HUVECs were generally focused on proinflammatory reactions, aberrant NO generation, and impaired vasodilatation. Therefore, the potential effects of serum fatty acids on blood clotting seem to be worthy of studying as they may contribute to serious consequences of obesity, including thrombosis [44] and cardiac complications [18]. Nowadays, particular interest is focused on vascular complications of COVID-19, especially in obese patients [45]. It was shown that a relatively higher risk of vascular complications and thrombosis occurs in those with elevated levels of palmitate but not unsaturated FAs in the plasma [46]. Furthermore, a higher risk of thrombosis in COVID-19 patients was attributed to an elevated vWF level [47]. Palmitate applied at a harmful concentration led to strong and reproducible inflammation and cellular stress response [9]. Contrary to this, relatively mild treatment of HUVECs with palmitate allows for changes that are not directly related to cell death but which characterise the adaptive response of endothelial cells to sub-critical stimuli. In consequence, we were balancing on the narrow edge between too-mild and non-effective palmitate treatment to strong and harmful conditions. A similar approach was applied in our previous studies on the mitochondrial metabolism of endothelial cells challenged by palmitate and TNF-α [13,48]. A precise analysis of *VWF* transcript revealed that 100 μM palmitate was sufficient to activate the vWF-encoding gene and doubling its concentration did not elevate this effect. Western blot analysis of cellular lysates showed that palmitate changes proportions between pro-vWF and mature vWF. Figure 4 suggests that palmitate influences vWF maturation, thereby increasing the amount of this protein ready to be secreted. Furthermore, 200 μM palmitate slightly increases the basal secretion of vWF and visibly enhances the secretory effects of histamine and forskolin. To point out the regulatory step which is responsible for this phenomenon, we have considered two possibilities: an accelerated maturation and secretion of vWF and/or a stimulated expression of its gene. However, the palmitate effect on mRNA encoding for selected proteins involved in vWF biology, including WPBs cargo, WPBs formation, vWF maturation, and processing was inconclusive, so the first possibility was not confirmed but not excluded either. vWF is the main but not the only cargo of WPBs as endothelial-specific secretory granules. P-selectin, CD63, and other inflammatory and angiogenic mediators are co-packaged into these structures [49]. Annexin A8 (AnxA8) is required for proper WPB maturation through the delivery of CD63 [50], an activator and regulator of P-selectin function [51]. Annexin A2 (AnxA2) participates in the late steps of stimulated WPB exocytosis [52,53]. ADAMTS13 is a vWF-cleaving metalloproteinase, regulating vWF multimers level in the circulation, thereby controlling thrombosis [54]. On the other hand, the stimulatory effect of palmitate on *VWF* gene expression seems to explain the observed increased vWF secretion. Thus, more vWF accumulated in cells and ready to be secreted may explain an apparent enhancement of the sensitivity of HUVECs to histamine or forskolin.

The regulation of the vWF encoding gene is controlled by a number of transcription factors such as ERG, GATA, SP1 [55], and NF-κB [56]. Palmitate-induced activation of NF-κB in C2C12 cells was previously demonstrated by other authors [57]. It was also suggested that this transcription factor plays a role in the regulation of the *VWF* gene [56,58]. As shown in Figure 7, a noticeable activation of NF-κB (visualised by increased p65 phosphorylation and a parallel decrease in IκBα levels) was observed 6 h after the addition of palmitate to the culture and persisted at least for 48 h. This suggests relatively slow palmitate-activated signal transduction in comparison with effects induced by LPS [59] but similar to that described by others [40].

The inhibitory effect of cardamonin on the palmitate-induced elevation of *VWF* transcript as shown in Figure 8 confirms the role of NF-κB in the regulation of *VWF*. In this experiment, HUVECs were treated with 100 μM palmitate. Under such conditions, any stimulatory effect on NF-κB activation visualised as increased p65 phosphorylation or IκBα level decrease was not observed, and a cellular level of adhesion proteins was not affected either. This apparent discrepancy may result from the fact that there are 12 physiological NF-κB dimers consisting of two out of five potential subunits: p50, p52, RelA (p65), cRel, and RelB [60]. Cardamonin is a poorly selective inhibitor of the NF-κB signalling pathway [61]; thus, an elevated level of phosphorylated p65, which is involved in the NF-κB canonical pathway as a p50/p65 heterodimer [62], is not a suitable marker of vWF-encoding gene activity, which is dependent on non-canonical NF-κB pathway [56].

Interestingly, in cells treated with 100 μM palmitate, the level of *TNF* transcript was unchanged, while it increased when cells were challenged with 200 μM palmitate. This activating effect was very strong after 6 h of the treatment and substantially smaller but still significant after 24 h. These data indicate that the effect of palmitate on the expression of the TNF-α-encoding gene depends not only on the concentration of this fatty acid, but also on the period of the treatment. To some extent, a similar conclusion was suggested by Kim et al. [63] for microglial cells treated with palmitate. On the other hand, this observation is opposed to numerous data evidencing that palmitate inevitably stimulates *TNF* gene expression [57] and the latter might be responsible for palmitate-evoked effects [64]. However, as shown in Figure 3, 100 μM palmitate elevates *VWF* transcript levels, while it is insufficient to activate the transcription of *TNF* (Figure 10); therefore, it seems that TNF-α is not involved in the stimulation of the vWF-encoding gene in response to palmitate in HUVECs.

Although the functional participation of TLR2 and TLR4 in cellular response to saturated fatty acids was well documented [20], the enormously strong elevation of cellular TLR2 protein levels in cells incubated with 200 μM palmitate is a new observation; however, it is in line with previously observed elevated *TLR2* transcript levels [65]. Our results suggest that palmitate specifically activates TLR4, but prolonged incubation with this FA elevated the total amount of TLR2. This is also in line with the activated NF-κB signalling pathway. It seems to be possible that the activation of TLR4 enhances TLR2 response through the NF-κB-dependent increasing expression of the TLR2-encoding gene [66]. In contrast, the effect of palmitate on the TLR4 protein level was very small and decreasing (Figure 9), possibly as a result of the ligand and receptor complex lysosomal degradation [67]. It is generally accepted that the stimulation of TLR2 and TLR4 results in elevated TNF-α synthesis [68]. We used changes in *TNF* mRNA levels as a marker of palmitate-induced signalling. The effect of palmitate on the *VWF* mRNA level increase, which is the main point of this study, is statistically significant but is less evident, and thus, the simultaneous increase in the *TNF* transcript level is easier to be followed. Similarly, as in the case of *VWF*, cardamonin reduces *TNF* gene expression regardless of the presence of palmitate. This confirms the role of NF-κB in the activation of both *TNF* and *VWF*.

The elevating effect of C29 on the *TNF* transcript suggests that the inhibition of TLR2 activates a compensatory response of alternative pathways, presumably involving TLR4. C29 induced the upregulation of TLR4-encoding gene and protein levels, which may be at least partially responsible for the observed effect. A similar phenomenon was reported in mice deficient in TLR2 or TLR4 [69,70]. In contrast, Jang et al. [35] found that the silencing of the *TLR2* gene resulted in a substantial reduction in the TNF-α level.

A reduced activity of TLR4 upon the treatment of cells with TAK-242 was accompanied by a decrease in TLR2 levels, possibly because of a downregulation of the NF-κB signalling pathway (see Figure 11). Co-incubation with C29 and TAK-242 together suppressed the stimulating effect of C29 on *TNF* transcription. It suggests that the cellular content of TLR2 is controlled by TLR4 activity via the NF-κB factor.

Incubation of HUVECs with palmitate in the presence of TAK-242, which disrupts TLR4 interaction with adaptor molecules [71], results in an upregulated expression of *CD36*, *TIRAP,* and *TLR6*. The latter may form a heterodimer with TLR2 upon stimulation, which recruits adaptor proteins involved in downstream signalling. Furthermore, in the presence of CD36, TLR6 forms a dimer with TLR4 [72]. CD36 acts as a co-receptor for both TLR2 [73] and TLR4 [74]. TIRAP and Myd88 are adaptor proteins necessary for TLR2 and TLR4 signal transduction [75].

The observed changes unveil an adaptive mechanism, which allows for maintaining signal transduction via the activation of alternative pathways. It was previously shown that overexpression of adaptor proteins, including MyD88 and TIRAP, diminished TAK-242 inhibitory effect [76]. The molecular bases behind such a cellular response are not clear for now and thus, further investigation seems to be important, especially in terms of possible C29 and TAK-242 effects in vivo. TAK-242 was shown to exert therapeutic effects in the animal model of sepsis [77]; however, results of the clinical trial were unsuccessful [78].

In contrast to TLR4 inhibition by TAK-242, the silencing of TLR4, although poorly noticeable at the protein level, was sufficient to elevate TLR2 content in cells 72 and 96 h after transfection with siRNA. On the other hand, the silencing of TLR2 resulted in a small and transient increase in TLR4 protein level. These observations are in line with previously published data, indicating functional and structural crosstalk between these receptors [69,70].

**Figure 15:**
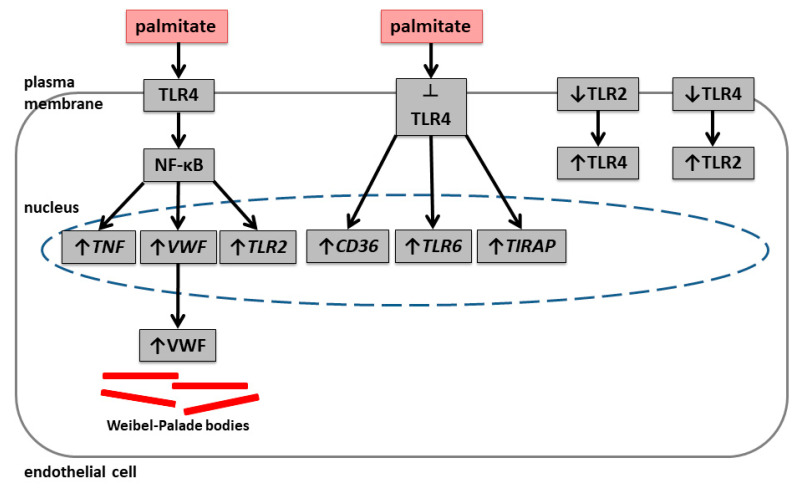
Schematic representation of palmitate effect on *VWF* gene expression and vWF maturation by NF-κB signalling, palmitate-induced upregulation of *CD36*, *TLR6*, and *TIRAP* as alternative signalling of inhibited TLR4, and compensatory effects between TLR2 and TLR4 in endothelial cells. TLR2 inhibition results in increased TLR4 activity and decrease of TLR2 protein level by silencing results in increased TLR4 protein level. Silencing of TLR4, even if it is only effective at the mRNA level and not the protein level, increases TLR2 protein levels.

The differences between the consequences of TLR4 inhibition and TLR4 silencing might result from differences in mechanism and therefore the time course of their action. The silencing of TLR4 resulted in a small decrease in TLR2 content after 48 h incubation with siRNA, which resembles the effect of TAK-242. Consequences of TLR inhibition by a decrease in activity and TLR gene silencing by a decrease in the mRNA or protein level may differ in molecular mechanisms, which ultimately needs further study. However, both approaches used confirmed mutual regulation between TLR2 and TLR4. Additionally, TLR4 inhibition revealed an activation of an alternative pathway in palmitate-treated cells. Figure 15 shows a schematic representation of the observed effects. 

To conclude, palmitate influences and elevates vWF secretion by endothelial cells and the mechanism of this phenomenon is related to an enhanced NF-κB signalling. Moreover, palmitate increases TLR2 protein level in HUVECs. The inhibition of TLR2 surprisingly increases *TNF* transcript levels probably because of the compensatory elevation of TLR4 activity. The latter response is blocked by TAK-242. C29 slightly increases TLR4 protein levels, while cellular TLR2 content is substantially reduced upon treatment of cells with TAK-242. The upregulation of proteins that mediate TLR4-induced response, including TLR6, CD36, and TIRAP, that was observed upon inhibition of TLR4, presumably reveals an adaptive mechanism allowing cells to maintain proper TLR4-dependent palmitate-induced signal transduction. The silencing of TLR2 (decreased protein and activity) results in a small increase in TLR4 level. On the other hand, the silencing of the TLR4 gene even if leading to a slightly reduced protein level results in a substantial elevation of TLR2 protein content without a corresponding effect on mRNA level. This suggests a post-transcriptional regulation. The translation of TLR-encoding mRNA is regulated by multiple miRNAs; thus, this could be a possible mechanism of the TLR protein level regulation [79]. We propose that there is an interplay between TLR2 and TLR4 that not only depends on the relative amount of these proteins itself but also their biological activity.

## 4. Materials and Methods

### 4.1. Cell Culture

Human umbilical vein endothelial cells (HUVECs) were purchased from American Type Culture Collection. Cells were cultured in EGM-2 medium containing 2% foetal bovine serum (Lonza, Basel, Switzerland) and used in passages 4–5. For all experiments, cells were cultured in collagen I (Sigma-Aldrich, Poznań, Poland)-coated plates based on previously described experiments [80]. Palmitate (PA) complexed with bovine serum albumin (BSA) was prepared according to the previously described method [81]. Cells were treated with 100 or 200 μM palmitate for 6, 24, or 48 h. Cells were preincubated for 1 h with inhibitors: cardamonin (CARD; C8249, Sigma-Aldrich), C29 (HY-100461, MedChemExpress, Monmouth Junction, NJ, USA), and TAK-242 (614316, Sigma-Aldrich, Poznań, Poland) before co-incubation with palmitate.

### 4.2. siRNA Transfection

Twenty-four hours after seeding, confluent cells were transfected with siRNA against TLR2 (siTLR2, siRNA ID: s168, 4427037, Thermo Fisher Scientific, Warsaw, Poland), TLR4 (siTLR4#1, siRNA ID s14194; siTLR4#2, siRNA ID s14195; 4427038, Thermo Fisher Scientific, Warsaw, Poland), and Silencer Select Negative Control No. 2 siRNA (siRNA neg; 4390847, Invitrogen, Thermo Fisher Scientific, Warsaw, Poland). Cells were transfected with a final concentration of 30 nM siRNA using jetPRIME transfection reagent (101000015, Polyplus-transfection, VWR, Gdańsk, Poland). After 24 h, medium was changed. Cells were analysed 48, 72, 96 h after transfection.

### 4.3. Cell Lysis and Western Blot Analysis

Cell lysates were prepared as previously described [82]. Protein concentration in lysates was determined using a Bradford protein assay (Bio-Rad, Warsaw, Poland). A total amount of 30 μg of protein per sample was separated via SDS-PAGE method under denaturing conditions on polyacrylamide gels and transferred into PVDF immobilon-E membrane (Millipore, Sigma-Aldrich, Poznań, Poland). Membranes were blocked 1 h at room temperature in 5% non-fat milk solution in 1× TBST, 0.001% Tween-20 (Sigma-Aldrich, Poznań, Poland) and incubated with primary antibodies specific for proteins of interest: ICAM-1 (sc-8439, Santa Cruz Biotechnology, Heidelberg, Germany), IκBα (#9242S, Cell Signaling Technology, Danvers, MA, USA), phospho-NF-κB p65 (Ser536) (#3033, Cell Signaling Technology, Danvers, MA, USA), TLR2 (A19125, ABclonal, Woburn, MA, USA), TLR4 (AF7017, Affinity Biosciences, Cincinnati, OH, USA), VCAM-1 (sc-13160, Santa Cruz Biotechnology, Heidelberg, Germany), vWF (ab6994, Abcam, Cambridge, UK), and vWFpp (11778-1-AP, Proteintech, Rosemont, IL, USA). Either β-actin (A3854, Sigma-Aldrich, Poznań, Poland) or β-tubulin (ab21058, Abcam, Cambridge, UK) was used as a housekeeping protein for normalisation. Secondary antibodies conjugated with horseradish peroxidase: anti-mouse (ab6728, Abcam, Cambridge, UK) or anti-rabbit (ab6721, Abcam, Cambridge, UK), and chemiluminescent substrate Immobilon Classico (WBLUC0500, Millipore Sigma-Aldrich, Poznań, Poland) was used for signal visualisation. Signal was detected using Fusion FX (Vilber Lourmat, Marne La Vallée, France). Densitometric analysis of specific bands was performed using BIO-1D software (Vilber Lourmat, Marne La Vallée, France).

Quantitative analysis of relative protein expression was normalised to β-actin or β-tubulin and compared to BSA treated as a control. Data are expressed as a percentage of appropriate BSA control and presented as mean ± SD.

### 4.4. RNA Extraction, Reverse Transcription, and Real-Time Quantitative PCR

RNA was isolated using TRI Reagent (T9424, Sigma-Aldrich, Poznań, Poland) and total RNA was extracted using the Total RNA Mini kit (031-100, A&A Biotechnology, Gdańsk, Poland) according to the manufacturer’s instructions. Concentration and quality of samples were measured using a NanoDrop spectrophotometer (Thermo Fisher Scientific, Warsaw, Poland). Complementary DNA (cDNA) was synthesised using First Strand cDNA Synthesis Kit (K1612, Thermo Fisher Scientific, Warsaw, Poland) and oligo(dT)_18_ primers from 1μg of total RNA.

Real-time qPCR reactions were performed using TaqMan Fast Universal PCR Master Mix (4352042, Applied Biosystems, Thermo Fisher Scientific, Warsaw, Poland) on a StepOnePlus Real-Time PCR System (Applied Biosystems, Thermo Fisher Scientific, Warsaw, Poland). Following TaqMan Gene Expression assays (4331182, Applied Biosystems, Thermo Fisher Scientific, Warsaw, Poland) were used for genes: *ACTB* (Hs01060665_g1), *ADAMTS13* (Hs00260148_m1), *ANXA2* (Hs00743063_s1), *ANXA8* (Hs04192999_gH), *CD36* (Hs00354519_m1), *CD63* (Hs01041238_g1), *MYD88* (Hs01573837_g1), *SELP* (Hs00927900_m1), *TIRAP* (Hs00364644_m1), *TLR2* (Hs02621280_s1), *TLR4* (Hs00152939_m1), *TLR6* (Hs01039989_s1), and *TNF* (Hs00174128_m1), *VWF* (Hs01109446_m1). To determine relative gene expression, *ACTB* was used for normalisation as the endogenous control. Relative quantification (RQ) for indicated gene expression was analysed via the 2^−ΔΔCT^ method using StepOne software v2.3.

### 4.5. von Willebrand Factor Level

To stimulate vWF secretion cells were treated with 100 or 200 μM palmitate for 48 h and subsequently incubated for 1 h in EBM-2 (Lonza, Basel, Switzerland) culture medium with 1 μM histamine or 10 μM forskolin. Cell culture supernatants were centrifuged at 2000× *g* for 10 min to remove debris. The von Willebrand factor level secreted to the culture medium was quantified using the Human von Willebrand Factor ELISA Kit (ab223864, Abcam, Cambridge, UK) according to the manufacturer’s instructions.

### 4.6. Apoptosis

Cells treated 48 h with 200 μM palmitate were trypsinized and apoptosis was assessed via flow cytometry using PE Annexin V Apoptosis Detection Kit I (559763, BD Pharmingen, Warsaw, Poland) according to the manufacturer’s instructions. Cells were washed with ice-cold PBS and resuspended in binding buffer. A total volume of 5 µL of PE Annexin V and 5 µL 7-AAD were added to each sample and incubated in the dark for 15 min at room temperature. Cells were treated for 24 or 48 h with 10 ng/mL TNF-α as a positive control. The fluorescence signals were measured using FACs Calibur (BD Biosciences, Warsaw, Poland) and analysed using CellQuest software (BD Biosciences, Warsaw, Poland).

### 4.7. Statistical Analysis

Analysis was performed using the GraphPad Prism 9. Experimental data were expressed as mean values ± standard deviation (SD). Data were analysed using Student’s unpaired two-tailed *t* test or via one-way ANOVA, followed by Dunnett’s multiple comparisons test for more than 2 groups (*p* < 0.05 was considered statistically significant).

## Data Availability

Data are contained within the article.
